# Magnetic Resonance Spectroscopy for Risk Stratification of Sonographically Indeterminate Ovarian Neoplasms: Preliminary Study

**DOI:** 10.3390/diagnostics11101847

**Published:** 2021-10-06

**Authors:** Yenpo Lin, Ching-Yi Hsieh, Yen-Ling Huang, Kueian Chen, Yu-Ting Huang, Ren-Chin Wu, Huei-Jean Huang, Hung-Hsueh Chou, Angel Chao, Chyong-Huey Lai, Gigin Lin

**Affiliations:** 1 Department of Medical Imaging and Intervention, Linkou Chang Gung Memorial Hospital, Chang Gung University, Taoyuan 33382, Taiwan; yenpojack@cgmh.org.tw (Y.L.); 9102091@cgmh.org.tw (Y.-L.H.); EGRIA0000@cgmh.org.tw (K.C.); m7131@adm.cgmh.org.tw (Y.-T.H.); 2 Clinical Metabolomics Core Laboratory, Linkou Chang Gung Memorial Hospital, Chang Gung University, Taoyuan 33382, Taiwan; chsieh2016@mail.cgu.edu.tw; 3 Department of Obstetrics and Gynecology and Gynecologic, Cancer Research Center, Linkou Chang Gung Memorial Hospital, Chang Gung University, Taoyuan 33382, Taiwan; qby@cgmh.org.tw (R.-C.W.); hjhuang@cgmh.org.tw (H.-J.H.); ma2012@cgmh.org.tw (H.-H.C.); angel945@cgmh.org.tw (A.C.); laich46@cgmh.org.tw (C.-H.L.); 4 Medical Imaging Research Center, Institute for Radiological Research, Chang Gung University, Taoyuan 33382, Taiwan; 5 Department of Medical Imaging and Radiological Sciences, Chang Gung University, Taoyuan 33382, Taiwan; 6 Department of Diagnostic Radiology, Keelung Chang Gung Memorial Hospital, Keelung 20401, Taiwan; 7 Department of Pathology, Linkou Chang Gung Memorial Hospital, Chang Gung University, Taoyuan 33382, Taiwan

**Keywords:** ovarian neoplasms, magnetic resonance imaging, magnetic resonance spectroscopy, diffusion-weighted imaging

## Abstract

We aim to assess the additional value of diffusion-weighted imaging (DWI) and magnetic resonance spectroscopy (MRS) for the risk stratification of sonographically indeterminate ovarian neoplasms. A total of 21 patients with diagnosed adnexal masses between 2014 and 2017 were divided into malignant (four serous cystadenocarcinomas, four endometrioid carcinomas, three clear cell carcinomas, and one carcinosarcoma) and benign (four cystadenomas, two teratomas, one fibroma, one endometrioma, and one corpus luteal cyst) groups. An apparent diffusion coefficient (ADC) value of 1.27 × 10^−3^ mm^2^/s was considered as the optimal threshold in distinguishing malignant from benign ovarian tumors (sensitivity and specificity: 100% and 77.8%, respectively). Choline peaks were detected in six of seven O-RADS (Ovarian-Adnexal Imaging-Reporting Data System) 4 lesions and corrected all of the DWI false-negative clear cell carcinoma. Based on the presence of the choline peaks, the diagnostic performance of MRS showed a sensitivity of 77.8%, a specificity of 100%, and an accuracy of 85.7%, respectively. In conclusion, MRS could potentially play a complementary role for DWI in tumor characterization, particularly for O-RADS 4 tumors or clear cell carcinomas.

## 1. Introduction

Ovarian cancer is the leading cause of death among gynecological malignancies [1]. Early diagnosis is challenging because ovarian cancer is asymptomatic until it reaches an advanced stage. The initial workup includes a physical examination, imaging, and an evaluation of tumor markers to differentiate between benign and malignant lesions. The best outcomes have been observed in patients whose primary treatment included the complete resection of all visible diseased tissue with optional combination chemotherapy [2]. Ultrasonography remains the primary tool for the noninvasive detection and initial evaluation of suspicious malignancies. Although the use of risk stratification systems—the Ovarian-Adnexal Imaging-Reporting Data System (O-RADS), have been proposed for providing standardized descriptors and consistent interpretations, 22% of ovarian lesions were reported to remain indeterminate in ultrasonographic imaging because the field of view and scanning windows are relatively limited [3].

Magnetic resonance imaging (MRI) has been proven to exhibit higher diagnostic accuracy than other imaging modalities in differentiating between malignant and benign lesions of the ovary [4]. MRI lexicon and scoring systems have been developed to optimize the characterization of adnexal lesions [5,6]. A recent multicenter cohort study using an MRI O-RADS score of 4 or 5, demonstrated a sensitivity of 93% and a specificity of 91% regarding the identification of malignant ovarian tumors [7]. However, these scoring systems require dynamic contrast-enhanced T1-weighted images for comparing perfusion curves between the myometrium and adnexal lesion, but the myometrial signal is not measurable in post-hysterectomy patients. Gadolinium-based MRI contrast agents are also not feasible for use in pregnant women or patients with impaired renal function [8]. Nonenhanced MRI techniques, such as diffusion-weighted imaging (DWI) and magnetic resonance spectroscopy (MRS), might add value to the use of MRI O-RADS. DWI measures the diffusivity of water molecules, which indicates the cellularity of lesions and potentially improves diagnostic confidence in the differentiation of malignant and benign ovarian lesions [4]. In clinical proton (^1^H)-MRS, detecting the total choline signal has demonstrated initial utility in characterizing cancers of the brain, prostate gland, and breast. MRS acquisition is feasible in normal ovaries and the solid part of tumor masses [9,10,11,12], but its clinical application for diagnosing ovarian tumors and making treatment decisions remains unexplored.

We aim to assess the additional value of DWI and MRS for the risk stratification of sonographically indeterminate ovarian neoplasms.

## 2. Materials and Methods

### 2.1. Patients

**Figure 1:**
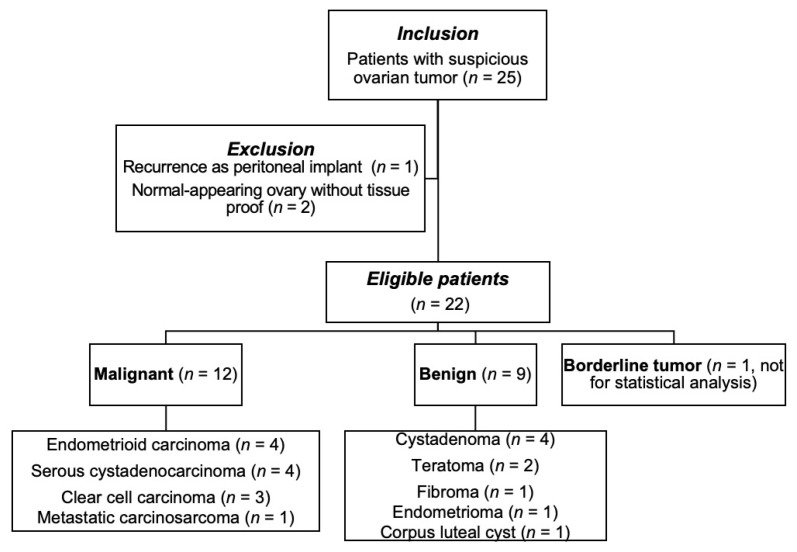
Flow diagram of patients and studies.

Our study was approved by the institutional review board of our hospital. The acquired data in this study was from another previously approved IRB protocol which was then re-analyzed for this study. (IRB 102-0620A3 and 103-7316A3). The need for informed consent was waived. This retrospective study was conducted in a tertiary referral center with a dedicated interdisciplinary gynecological oncology team. From April 2014 to July 2017, the gynecological MR examination data of 25 consecutive patients with indeterminate adnexal tumor(s) recorded using gynecological ultrasound were enrolled (Figure 1). The inclusion criteria for this study were: (1) patients with clinical suspicion of ovarian tumors but unable to determine their nature using ultrasound. The exclusion criteria were: (1) clinically apparent infectious or inflammatory etiology; (2) recurrence as a peritoneal implant; and (3) lesions without histopathological tissue proof. We dichotomized the tumors into malignant and benign groups. The clinical parameters of tumor histology, differentiation, the presence of high levels of tumor marker CA-125, clinical International Federation of Gynecology and Obstetrics (FIGO) stage, and major methods of treatment were recorded.

### 2.2. MRI Scan

Enrolled patients were imaged using a 3 Tesla (T) MR system (Siemens, Erlangen, Germany). The lower nine elements of the integrated spine coil and the lower six elements of the body-phased array coil were used to study the entire pelvis. T2-weighted (T2W; repetition time ms/echo time ms (TR/TE), 5630/87; average, 3; matrix, 256 × 320; field of view (FOV 20 × 20 cm^2^), 20 cm) and DWI using a single-shot echo-planar technique with fat suppression (TR/TE, 3300/79; average, 4; section thickness, 4 mm; gap 1 mm; matrix, 128 × 128; FOV 20 × 20 cm^2^) were performed. The DW gradients were applied orthogonally in slice-selective, phase encoding, and readout directions. Apparent diffusion coefficient (ADC) maps were generated using isotropic DWI with *b* values of 0 and 1000 s/mm^2^, by calculating the slope of the logarithmic decay curve of signal intensity against b values (Syngo, Siemens, Erlangen, Germany). Sequences were obtained with identical slice thickness and gaps in the axial and sagittal planes in T2W and DWI to cover the entire true pelvis. Intravenous bolus injection of 0.1 mmol/kg bodyweight of contrast medium (gadopentetate dimeglumine, Magnevist, Schering, Berlin, Germany) was administered after the spectroscopy scan. According to the white paper proposed by the American College of Radiology on Ovarian-Adnexal Reporting Lexicon for MRI, the recommended method for assessing enhancement patterns is dynamic contrast enhancement (DCE) MRI. Alternatively, a nondynamic contrast MR acquisition is acquired precontrast and at 30 to 40 s after the contrast injection. In this study, we performed a nondynamic evaluation of the mass. The patients were encouraged to control their free-breathing and minimize it during MR examinations. No premedication was administered. 

### 2.3. MR Spectroscopy Acquisition

We used triplane localizer 1D MR spectroscopy with point-resolved spectroscopy (PRESS), with the volume of interest (VOI) 10 × 20 × 20 mm^3^ prescribed by gynecological radiologists (Y.T.H. or G.L.) being completely placed within the solid part of the ovarian lesion. We optimized the following parameters for PRESS: TR/TE, 2000/35; 128 averages; vector size, 1024 points; bandwidth, 1200 Hz [13]. The VOI was applied water suppression and six outer volume suppression bands to suppress lipid contamination with advanced auto shimming. In addition, non-water suppressed spectra were displayed as concentration references, four averages, with a total scan time of 37.4 s. MRS was conducted without any patient discomfort or adverse events being reported. 

### 2.4. MRI Analysis

Two radiologists (G.L and Y.L, with 15 and 4 years of experience in gynecological radiology, respectively) evaluated the size, conventional MR features, functional MR features, including DW hyperintensities and mean ADC value of the solid portions of the tumor masses, and spectroscopic results. To resolve interobserver discrepancies, a consensus was reached after discussion. MR O-RADS scores were assigned using a modified enhancement evaluation based on postcontrast T1-weighted (T1W) images. A visual qualitative assessment compared the mass enhancement with the myometrium without the region of interest (ROI) [7,14]. The scores were assigned based on the following: (1) no adnexal mass; (2) benign mass—adnexal unilocular cyst with simple fluid and no solid tissue, adnexal unilocular cyst with endometriotic fluid and no internal enhancement, adnexal cyst with fatty content (unilocular or multilocular) and no solid tissue, no wall enhancement or adnexal lesion with homogeneous T2W hypointensity, and solid tissue in high *b*-value DWI; (3) probably benign mass—adnexal unilocular cyst with proteinaceous or hemorrhagic fluid and no solid tissue or adnexal multilocular cyst and no solid tissue; (4) indeterminate mass—adnexal lesion with solid tissue enhancement that is less than that of the outer myometrium; and (5) probably malignant mass—adnexal lesion with solid tissue enhancement greater than or equal to that of the outer myometrium or peritoneal/omental implants. DWI hyperintensity was deemed to be present if the lesion had a signal that was more than 50% higher than that of the outer myometrium in high b-value DWI (*b* = 1000 s/mm^2^) and a lower signal on ADC maps. The ADC values of each primary tumor were measured using manually drawn ROIs within the solid part of the main tumors, which were identified using T1W and T2W images. The ADC values measured independently by the two readers were averaged and were used as representative ADC values for each tumor. T1W hyperintensity was defined as the presence of any mass area with a higher signal than the fatty bone marrow of the pubic symphysis. T2W hyperintensity was defined as more than half of the mass exhibiting a signal that was higher than that of the outer myometrium on the T2W images.

### 2.5. MR Spectroscopy Analysis

Suppressed and unsuppressed water data were analyzed by choosing the “tumor” basis in the LCModel software (v. 6.3–0 K; Provencher, Ontario, CA, Canada) on a Linux workstation, which applied a linear combination of multiple spectra. Typically, all of the data in the analysis range of 4.0–1.0 ppm are used in a constrained least-squares analysis to fit the model parameters (metabolite concentrations, phases, referencing shift, line shape, baseline, etc.) and estimates of the goodness-of-fit using the Cramer–Rao lower bound (CRLB) [15]. Our spectra criteria are as follows: MR spectra were excluded if the CRLB exceeded 20% for creatine (δ 3.0 and 3.9 ppm), choline (δ 3.2 ppm), lipid methyl (δ 0.9 ppm), and lipid methylene (δ 1.3 ppm) and 30% for unsaturated lipids (δ 2.0 ppm). A metabolite peak was considered twofold higher than the average noise level. The signal to noise ratio (SNR) of the water-suppressed MR spectra is defined as the ratio of the maximum in the spectrum-minus-baseline over the analysis window to twice the root mean square (RMS) residuals from a peak-free region of the spectrum. Linewidth is defined as the full width at half maximum (FWHM) of a singlet resonance measured in the frequency domain in ppm at half maximum height.

### 2.6. Pathological Diagnoses

Final diagnoses were made by a pathological examination of surgical specimens. We further divided the patients into malignant and benign groups according to the pathological diagnoses of the tumors.

### 2.7. Statistical Analysis

Data were analyzed using MedCalc for Windows, V. 9.2.0.0 (MedCalc Software, Mariakerke, Belgium). Data were not normally distributed based on the Kolmogorov–Smirnov test. Continuous variables were analyzed using the Wilcoxon signed-rank test and Mann–Whitney *U* test (two-group comparisons). Pearson’s chi-square test was used to evaluate categorical variables. Sensitivity, specificity, and diagnostic accuracy were represented using 95% confidence intervals. Areas under the receiver operating characteristic (ROC) curve (AUROCs) were calculated to compare diagnostic performance in each group. Values of *p* < 0.05 indicated significant differences. A Bonferroni post hoc correction was conducted to reduce Type I error by dividing the original α-value regression analyses.

## 3. Results

### 3.1. Patient Demographics and Pathological Diagnoses

The flowchart of the study is illustrated in Figure 1.

**Figure 2:**
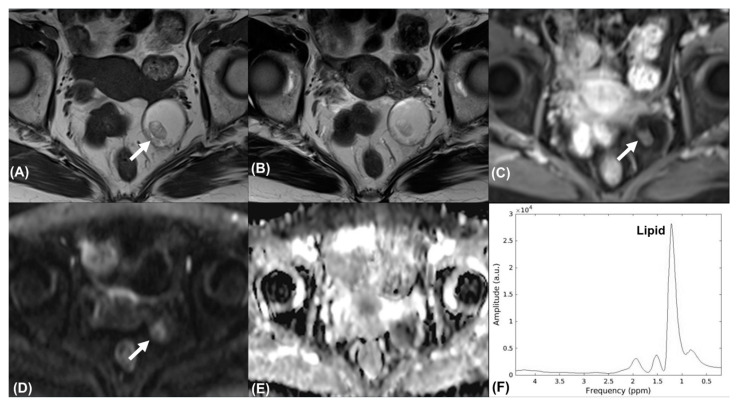
Mature cystic teratoma in a 67-year-old woman. Axial (**A**) T1-weighted, (**B**) T2-weighted, and (**C**) contrast-enhanced T1-weighted MR images show a left adnexal cystic mass with fatty content. Axial (**D**) high-*b*-value DW image (*b* = 1000 s/mm^2^) and (**E**) ADC map demonstrated mild restricted diffusion of the solid nodule (*arrow*). The tumor was classified as an O-RADS 3 lesion. (**F**) MR spectroscopy depicts prominent resonances corresponding to lipids (demonstrated using the FID-A toolkit [16]).

**Figure 3:**
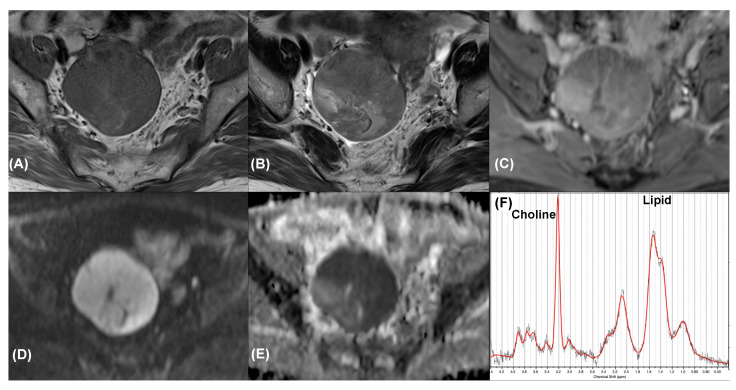
Synchronous endometrioid cell carcinoma in a 55-year-old woman with endometrial cancer. Axial (**A**) T1-weighted, (**B**) T2-weighted, and (**C**) contrast-enhanced T1-weighted MR images show a right adnexal tumor with solid portion enhancement. Axial (**D**) high-*b*-value DW image (*b* = 1000 s/mm^2^) and (**E**) ADC map demonstrated marked restricted diffusion of the tumor. The tumor was classified as an O-RADS 4 lesion. (**F**) MR spectroscopy depicts choline and lipid peaks (SNR = 20.0 ± 1.8; linewidth = 8.2 ± 1.2 Hz). Data are in black, and the corresponding fit is in red.

**Figure 4:**
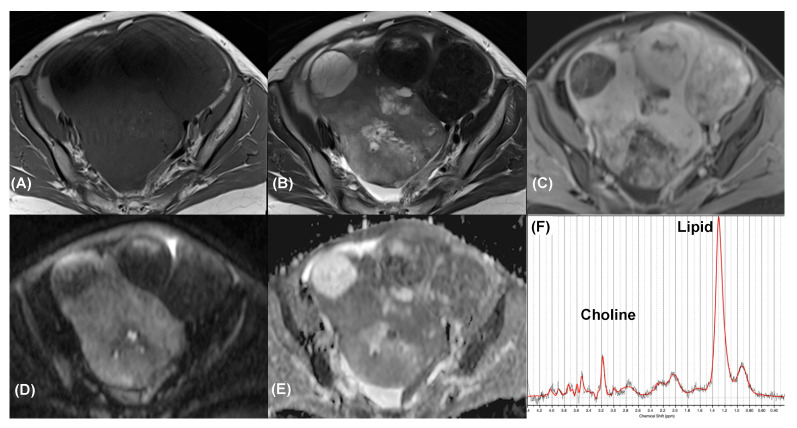
Clear cell carcinoma in a 33-year-old woman. Axial (**A**) T1-weighted, (**B**) T2-weighted, and (**C**) contrast-enhanced T1-weighted MR images show a cystic tumor in adnexa with irregular solid component (*arrow)* exhibit marked contrast enhancement. Axial (**D**) high-*b*-value DW image (*b* = 1000 s/mm^2^) and (**E**) ADC map demonstrated marked restricted diffusion of the solid component. The tumor was classified as an O-RADS 5 lesion. (**F**) MR spectroscopy depicts choline and lipid peaks (SNR = 29.0 ± 1.8; linewidth = 4.7 ± 1.2 Hz). Data are in black, and the corresponding fit is in red.

Overall, 21 patients were eligible for final analysis (age range, 41–67 years; median, 41.5 years), with patient the demographics summarized in Table 1. The benign group comprised four cystadenomas, two teratomas (Figure 2), one fibroma, one endometrioma, and one corpus luteal cyst. The malignant group comprised four serous cystadenocarcinomas, four endometrioid carcinomas (Figure 3), three clear cell carcinomas (Figure 4), and one carcinosarcoma.

### 3.2. Morphological MR Features (Modified O-RADS)

The O-RADS scores were ≥4 for the malignant lesions and <4 for the benign lesions, respectively. Significant differences in all of the parameters considered in the O-RADS scores, namely morphology, external contour, internal wall, internal septa, and solid part with enhancement, were observed between the malignant and benign masses (*p* < 0.05 for all), as summarized in Table 2. Using a cutoff value of an O-RADS score of 4, we demonstrated a positive predictive value of 100% in our series. All of the malignant tumors (Figure 3 and Figure 4) exhibited the morphology of multiloculated cystic and solid tissue (the solid part with enhancement, papillary projection, mural nodule, or irregular septation) with contrast enhancement (100%, *p* < 0.005). Multiloculated purely cystic morphology was the most common feature among the benign tumors (44.4%). The mean size of the tumors in the malignant group (mean ± standard deviation, 7.9 ± 5.2 cm) was larger than that in the benign group (7.4 ± 4.3 cm), although the difference in size was not statistically significant (*p* = 0.80). The AUROC of the MRI O-RADS was 1000.

### 3.3. Value Addition of DWI and ADC Value in Tumor Classification

**Figure 5:**
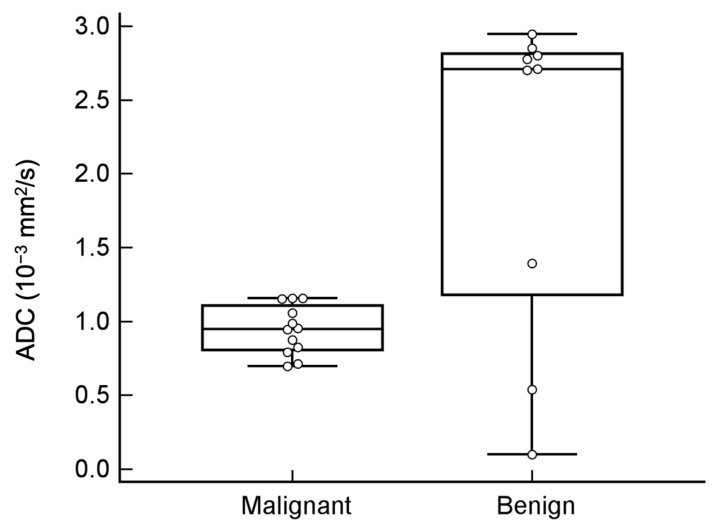
ADC values of tumors and areas under the receiver operating characteristic (ROC) curve (AUROCs). The ADC value of malignant tumors was significantly lower than that of benign tumors but with a remarkable overlap. Using an ADC value of ≤1.27 × 10^−3^ mm^2^/s to distinguish malignant from benign ovarian tumors with sensitivity and specificity were 100% and 77.8%, respectively.

The DWI hyperintensity of the solid part was observed in all of the malignant tumors; however, DWI hyperintensity was only noted in two teratomas (Figure 2) and one fibroma in the benign group (100% vs. 33%, *p* = 0.002). The mean ADC value was significantly lower in the malignant group than in the benign group (0.94 vs. 2.09 × 10^−3^ mm^2^/s, *p* = 0.034). The ADC values of the two teratomas were 0.54 × 10^−3^ mm^2^/s and 0.99 × 10^−3^ mm^2^/s, and the ADC value of the fibroma was 1.39 × 10^−3^ mm^2^/s. The other benign tumors showed no overlapping features with the malignant lesions. The ROC curve (Figure 5) indicated that an ADC value of 1.27 × 10^−3^ mm^2^/s was an optimal threshold for distinguishing between malignant and benign ovarian tumors (sensitivity and specificity: 100% and 77.8%, respectively). The AUROC of DWI was 0.722, which was significantly less than that of MRI O-RADS (*p* = 0.007).

### 3.4. Additional Value of MRS

In total, 14 masses (nine malignant and five benign) were analyzed through MRS in the preoperative MRI. In this study, choline peaks were detected in seven of the nine malignant tumors (78%) but were not detected in any of the benign tumors (*p* = 0.021). Using the choline peak detected in MRS suggested malignant pathology in seven masses; seven of them were true malignant (TP), and no benign mass was faultily diagnosed as malignant (FP). MRS also suggested benign pathology in five cases; all of them were true benign (TN), but two malignant masses (an endometrioid carcinoma and a serous cystadenocarcinoma) did not exhibit detectable choline peaks (FN). Based on the presence of a choline peak, diagnostic performance values along a 95% confidence interval were calculated with a sensitivity of 77.8% (45.3–96.0%), a specificity of 100% (56.6–100%), and an accuracy of 85.7% (57.2–98.2%). Notably, all of the masses with a choline peak simultaneously exhibited a lipid peak. Elevated lipid resonance was observed in all benign tumors except for the fibroma. The AUROC of MRS was 0.792, which was significantly lower than that of MRI O-RADS (*p* = 0.005). None of the quantitative MRS measurements, including creatine, choline, and lipid resonances at the 0.9 ppm, 1.3 ppm, and 2.0 ppm, demonstrated statistically significant differences between the benign and malignant groups. The complementary role of DWI and MRS in characterizing malignant versus benign ovarian tumors is shown in Table 3. Choline peaks were detected in six of seven O-RADS 4 lesions and corrected all of the DWI false-negative clear cell carcinoma.

### 3.5. Borderline Tumor

The only borderline tumor in our series showed a multiloculated cystic pattern with a solid enhancement and was classified as an O-RADS 4 lesion. It demonstrated hyperintensity on DWI, and the mean ADC value of the solid part was 1.31 × 10^−3^ mm^2^/s. Elevated lipid resonance was observed in MRS, but no choline peak was observed. There was no extraovarian involvement.

## 4. Discussion

The MRI O-RADS scoring system is derived from the ADNEX MR scoring system [7], and according to published literature, a score ≥ 4 suggests the need for prompt surgery [17,18]. Morphological features (modified O-RADS), namely morphology, external contour, internal wall, internal septa, and solid part with enhancement, demonstrated an excellent AUROC of 1000 in the present study. However, the O-RADS is reader-dependent and might be subjective. Quantitative ADC measurements were helpful in terms of objective diagnostics with an AUROC of 0.722, which might be helpful for readers with less experience. Choline peaks on the MRS were detected in six of seven O-RADS 4 lesions and could be applied to correct DWI false negativity. Our data were consistent with the findings of Sasaguri et al. [17], who showed that a practical approach to evaluating adnexal masses using O-RADS may start with the most high-risk characteristics—for example, a peritoneal implant, solid tissue of the mass, a papillary projection, a mural nodule, irregular septation, or a solid enhancing portion. In our series, all of the masses with peritoneal implants were malignant and were categorized as O-RADS 5, and the absence of solid tissue was considered to indicate a high probability of benignity [5]. Only the fibroma in our series specifically exhibited an O-RADS score of 2 and was a solid enhancing lesion with homogeneous T2W hypointensity and solid tissue with a high b-value DWI [19]. Heterogeneous enhancement might be observed in fibromas larger than 6 cm [20] and should be correlated with other imaging sequences. Overall, O-RADS yields accurate risk stratification based on morphological appearance and certain functional MR techniques, but the positive predictive value of O-RADS 4 is 5–95% according to Sadowski et al. [6], which indicates the need for more precise assessment. Furthermore, the scoring of some tumors, such as borderline tumors, may be difficult if using the O-RADS scale.

Our study identified a significant correlation between DWI hyperintensity and malignant masses, which is consistent with previous reports that have used b values of 0 and 1000 s/mm^2^ [21,22]. For the fibroma, a benign ovarian tumor with limited specificity was noted because of dense cellularity that resulted in restricted diffusion on DWI [23]. Several other benign female pelvic tumors, chiefly endometriomas, dermoid cysts, and fibroids, can also exhibit restricted diffusion [24]. Even normal ovaries may exhibit restricted diffusion, and thus, image interpretation in conjunction with the results of other image sequences is essential. The probable cutoff value of the ADC value to differentiate between malignant and benign adnexal masses was reported to be 1.13 × 10^−3^ mm^2^/s in previous literature [25,26] and 1.27 × 10^−3^ mm^2^/s in our study. However, the ADC values of two teratomas (0.54 × 10^−3^ mm^2^/s and 0.99 × 10^−3^ mm^2^/s, respectively) were lower than these cutoff values and were found to be lower than those of malignant masses in our study; these results are comparable with those of Kim et al. [22], which might explain the high sensitivity but low specificity of using ADC cutoff values alone as an indicator of malignancy. Hence, the diagnoses of teratomas or fibromas using solely DWI should be interpreted cautiously.

In our series, choline peaks were specifically detected in seven of nine malignant masses and were not detected in any of the benign masses. Choline is a cell membrane marker that exhibits alteration and accumulation in cancer cells. Detection of a choline peak or an increased choline-to-creatine ratio has been reported in ovarian cancers [11,27,28,29,30]. Having information concerning elevated choline peaks increased our confidence in diagnosing a mass as a malignancy. Our preliminary findings suggested that MRS might have a complementary role for DWI, in upgrading O-RADS categorization, to correct DWI false negativity, and to achieve a more precise preoperative diagnosis. Similar findings were also reported by Mansour et al. [9], who stated that MRS had a negative predictive value compared to dynamic contrast-enhanced MRI (90.0% vs. 88.9%). However, the sensitivity (75.7%) reported by Mansour et al. was considerably lower than that in our study (100%), where only the visual assessment of choline peak was used. A lipid peak was also observed in lesions classified as O-RADS 2 and 3 in benign masses. However, the elevated choline resonance on MRS should be cautiously interpreted because it might also be detected in benign tumors due to substantial proliferative activity; no specific cutoff value has been reported yet [27]. 

Accurate patient selection and surgical planning are mandatory before laparoscopic surgery to optimize risk stratification for women with ovarian neoplasms in order to balance the risks of intraoperative spillage against the benefit of a minimally invasive approach [31]. Borderline tumors are commonly staged as malignant tumors, and accurate preoperative MR images could prevent unnecessary complete surgical staging, particularly in patients who could benefit from potential fertility-sparing measures. The only borderline tumor observed in our series followed the principle of predominantly cystic morphology, a regular thin wall, and a lack of extraovarian disease, but the solid enhancing portion and was classified as an O-RADS 4 lesion. The mean ADC value of the solid part (1.31 × 10^−3^ mm^2^/s) and no choline peak on MRS suggested it belonging to a benign entity, but the elevated lipid resonance raised the suspicion of the borderline tumor. In line with our study, Ma et al. [32] also reported that the mean choline-to-creatine ratios were significantly lower in borderline tumors than in malignant epithelial ovarian tumors. The value of MRS in the differentiation of borderline and malignant ovarian tumors warrants further investigation.

## 5. Limitations

The present study had some limitations. First, although we tried to include all of the different tumor varieties in addition to epithelial cancers and carefully selected non-inflammatory or non-infectious etiology, the sample size was still small. Our study cohort represented a real-life clinical situation in which tumors confined within the pelvis could not be classified through ultrasonography unless the disease was at an advanced stage. Second, overlapping DWI features and pitfalls were identified, such as water restriction in normal tissue (premenopausal uterine endometrium and ovarian mesenchyma), highly cellular benign tumors, and minimal restriction in some well-differentiated tumors. Heterogeneous ovarian tumors with mixed solid and cystic components may cause difficulty in ADC value measurement. Third, although the advantage of MRS includes the ability to evaluate multiple metabolites in most commercial MR scanners, its clinical value depends on metabolite peaks being clearly resolved and biochemically relevant. The quantification of an absolute value may have potential difficulties such as spatial variation in the transmission and the reception profiles of the coils and motion for both DWI and MRS. Compared to DWI, MRS is relatively less familiar to clinicians. Our results certainly require confirmation by conducting additional studies with a larger number of cases to obtain a generalizable result.

## 6. Conclusions

MRI O-RADS is useful to identify ovarian malignancy, yet it is also subjective and reader dependent. Quantitative ADC measurement is objective and might be helpful for readers with less experience. Choline peaks on MRS could potentially play a complementary role for DWI in tumor characterization, particularly for O-RADS 4 tumors or clear cell carcinomas. Optimizing choline measurement on MRS might lead to improvements in differentiating between benign and malignant ovarian tumors.

## Figures and Tables

**Table 1:** Demographics of the study participants.

	Malignant		Benign	
Total number	12		9	
Age, median (y)	48 (33–84)		41 (17–67)	
Tumor size, mean (cm)	6.9 (3.2–22)		5.9 (4.3–17.7)	
Histopathology	Endometrioid carcinoma Clear cell carcinoma Serous cystadenocarcinoma Metastatic carcinosarcoma	4 3 4 1	Teratoma Cystadenoma Fibroma Endometrioma Corpus luteal cyst	2 4 1 1 1
Menopausal status				
Premenopausal	6 (50%)		6 (66.7%)	
Postmenopausal	6 (50%)		3 (33.3%)	
FIGO stage	IA	6		
	IC	4		
	III	2		
Treatment			9 (100%)	
Surgery alone	6 (50%)			
Surgery with adjuvant treatment	6 (50%)			

Unless otherwise indicated, data are the number of patients, with the range in parentheses.

**Table 2:** MR imaging characteristics between malignant and benign ovarian tumors.

	Malignant (*n* = 12)			Benign (*n* = 9)			*p*-Value
Size (cm)	7.9	±	5.2	7.4	±	4.3	0.808
Mean ADC (10^−3^ mm^2^/s)	0.9	±	0.2	2.1	±	1.1	0.034 *
Bilaterality							0.553
No	11		91.7%	7		77.8%	
Yes	1		8.3%	2		22.2%	
Morphology							<0.001 *
No	0		0.0%	8		88.9%	
Yes	12		100.0%	1		11.1%	
External contour							<0.001 *
No	0		0.0%	9		100.0%	
Yes	12		100.0%	0		0.0%	
Internal wall							<0.001 *
No	0		0.0%	9		100.0%	
Yes	12		100.0%	0		0.0%	
Internal septa							<0.001 *
No	2		16.7%	9		100.0%	
Yes	10		83.3%	0		0.0%	
Solid par							<0.001 *
No	0		0.0%	7		77.8%	
Yes	12		100.0%	2		22.2%	
Enhancement of solid part							<0.001 *
No	1		8.3%	8		88.9%	
Yes	11		91.7%	1		11.1%	
T1W							0.021 *
No	0		0.0%	4		44.4%	
Yes	12		100.0%	5		55.6%	
T2W							<0.001 *
No	1		8.3%	8		88.9%	
Yes	11		91.7%	1		11.1%	
DWI							0.002 *
No	0		0.0%	6		66.7%	
Yes	12		100.0%	3		33.3%	
Extraovarian lesion							0.367
No	6		50.0%	7		77.8%	
Yes	6		50.0%	2		22.2%	

* The differences were significant according to Bonferroni’s correction for multiple comparisons. Unless otherwise indicated, data are presented as mean ± standard deviation. ADC = apparent diffusion coefficient; T1W = T1-weighted image; T2W = T2-weighted image; DWI = T1-weighted image.

**Table 3:** The complementary role of DWI and MRS in characterizing malignant vs. benign ovarian tumors.

Case	Histopathology	Malignancy	O-RADS	DWI	MRS
1	Serous cystadenocarcinoma	Yes	5	TP	FN
2	Serous cystadenocarcinoma	Yes	5	TP	FN
3	Metastatic carcinosarcoma	Yes	5	TP	FN
4	Clear cell carcinoma	Yes	5	FN	TP *
5	Clear cell carcinoma	Yes	4	FN	TP *
6	Clear cell carcinoma	Yes	4	FN	TP *
7	Endometrioid carcinoma	Yes	4	FN	TP *
8	Endometrioid carcinoma	Yes	4	TP	FN
9	Endometrioid carcinoma	Yes	4	TP	TP
10	Endometrioid carcinoma	Yes	4	TP	TP
11	Serous cystadenocarcinoma	Yes	4	TP	TP
12	Serous cystadenocarcinoma	Yes	4	TP	FN
13	Serous cystadenoma	No	3	TN	TN
14	Mucinous cystadenoma	No	3	TN	TN
15	Mucinous cystadenoma	No	3	TN	TN
16	Serous cystadenoma	No	3	TN	TN
17	Teratoma	No	3	FP	TN
18	Teratoma	No	2	FP	TN
19	Endometrioma	No	2	TN	TN
20	Fibroma	No	2	TN	TN
21	Corpus luteal cyst	No	1	TN	TN

TP, true positive; TN, true negative; FP, false positive; FN, false negative; O-RADS, Ovarian-Adnexal Imaging-Reporting Data System; DWI, diffusion-weighted imaging; MRS, magnetic resonance spectroscopy. * DWI false negativity corrected by MRS criteria.

## Data Availability

Data of this study will be available upon request.
